# SjAPI, the First Functionally Characterized *Ascaris*-Type Protease Inhibitor from Animal Venoms

**DOI:** 10.1371/journal.pone.0057529

**Published:** 2013-03-22

**Authors:** Zongyun Chen, Bin Wang, Jun Hu, Weishan Yang, Zhijian Cao, Renxi Zhuo, Wenxin Li, Yingliang Wu

**Affiliations:** 1 State Key Laboratory of Virology, College of Life Sciences, Wuhan University, Wuhan, China; 2 Key Laboratory of Biomedical Polymers of Ministry of Education, Department of Chemistry, Wuhan University, Wuhan, China; Instituto Butantan, Brazil

## Abstract

**Background:**

Serine protease inhibitors act as modulators of serine proteases, playing important roles in protecting animal toxin peptides from degradation. However, all known serine protease inhibitors discovered thus far from animal venom belong to the *Kunitz*-type subfamily, and whether there are other novel types of protease inhibitors in animal venom remains unclear.

**Principal Findings:**

Here, by screening scorpion venom gland cDNA libraries, we identified the first *Ascaris*-type animal toxin family, which contains four members: *Scorpiops jendeki Ascaris*-type protease inhibitor (SjAPI), *Scorpiops jendeki Ascaris*-type protease inhibitor 2 (SjAPI-2), *Chaerilus tricostatus Ascaris-type protease inhibitor* (CtAPI), and *Buthus martensii Ascaris-type protease inhibitor* (BmAPI). The detailed characterization of *Ascaris*-type peptide SjAPI from the venom gland of scorpion *Scorpiops jendeki* was carried out. The mature peptide of SjAPI contains 64 residues and possesses a classical *Ascaris*-type cysteine framework reticulated by five disulfide bridges, different from all known protease inhibitors from venomous animals. Enzyme and inhibitor reaction kinetics experiments showed that recombinant SjAPI was a dual function peptide with α-chymotrypsin- and elastase-inhibiting properties. Recombinant SjAPI inhibited α-chymotrypsin with a Ki of 97.1 nM and elastase with a Ki of 3.7 μM, respectively. Bioinformatics analyses and chimera experiments indicated that SjAPI contained the unique short side chain functional residues “AAV” and might be a useful template to produce new serine protease inhibitors.

**Conclusions/Significance:**

To our knowledge, SjAPI is the first functionally characterized animal toxin peptide with an *Ascaris*-type fold. The structural and functional diversity of animal toxins with protease-inhibiting properties suggested that bioactive peptides from animal venom glands might be a new source of protease inhibitors, which will accelerate the development of diagnostic and therapeutic agents for human diseases that target diverse proteases.

## Introduction

Serine protease inhibitors are of broad interest and can act as tools and modulators for serine proteases [1]. Diverse interactions between these inhibitors and proteases play key roles in a variety of physiological functions, such as blood coagulation, fibrinolysis, apoptosis, development, inflammation, and complement activation in humans [2]–[4]. Serine protease inhibitors exist widely, not only in the bodies of various animals, but also in the secretions of parasites, hematophagous invertebrates, amphibian skins, and the venom glands of poisonous animals [1], [5]–[7]. Previous studies have indicated that protease inhibitors from venom glands might protect toxin peptides from degradation by various proteases and play important roles for venomous animal survival [8]–[10]. All known serine protease inhibitors discovered thus far from animal venom belong to the *Kunitz*-type subfamily; whether there are other novel types of protease inhibitors in animal venom remains unclear [11].

*Ascaris*-type peptides constitute an important class of serine protease inhibitors. The *Ascaris*-type peptide was almost isolated from the parasitic worm *Ascaris suum* and has been implicated in the survival of the parasite within the host by the inhibition of exogenous host proteases [12]–[14]. Almost all *Ascaris*-type peptides have a common structural characteristic with four short β-strands arranged in two approximately perpendicular β-sheets and stabilized by five disulfide bridges: Cys I–Cys VII, Cys II–Cys VI, Cys III–Cys V, Cys IV–Cys X, and Cys VIII–Cys IX [15]. The reactive site loop of *Ascaris*-type peptides is almost bounded by two disulfide bridges (Cys II–Cys VI and Cys III–Cys V) and is part of the long loop connecting strands β1 and β2, as seen in the *Ascaris* sp. trypsin inhibitor (ATI), *Apis mellifera* cathepsin G/chymotrypsin inhibitor-1 (AMCI-1), chymotrypsin/elastase inhibitor-1 (C/E-1), and *Bombina bombina* skin trypsin inhibitor (BSTI) [16]–[18].

Here, by searching scorpion cDNA libraries, we reported four *Ascaris*-type peptide genes encoding SjAPI (*Scorpiops jendeki Ascaris*-type protease inhibitor), SjAPI-2 (*Scorpiops jendeki Ascaris*-type protease inhibitor 2), CtAPI (*Chaerilus tricostatus Ascaris-type protease inhibitor*), and BmAPI (*Buthus martensii Ascaris-type protease inhibitor*), and the detailed characterization of *Ascaris*-type peptide SjAPI from the venom gland of the scorpion *Scorpiops jendeki*. Enzyme and inhibitor reaction kinetics experiments showed that SjAPI was a dual-functional peptide with α-chymotrypsin- and elastase-inhibiting properties. To our knowledge, SjAPI is the first functionally characterized *Ascaris*-type toxin peptide derived from animal venom glands [11], [19].

## Materials and Methods

### Data screening for *Ascaris*-type animal toxins

The venom gland cDNA libraries of various scorpions from China were constructed as described previously [20], [21]. Random colonies were selected for sequencing using the ABI 3730 automated sequencer. Open reading frames (ORFs) of the sequences were characterized using ORFfinder (http://www.ncbi.nlm.nih.gov/projects/gorf/). Signal peptides were removed using the SignalP 4.0 Server [22]. All sequence alignments were performed using Clustal_X 1.83 software followed by manual adjustment. Sequences of *Ascaris*-type toxins were obtained by searching against our own cDNA libraries and the GenBank National Center for Biotechnology Information database (http://www.ncbi.nlm.nih.gov/) using the Basic Local Alignment Search Tool algorithm.

### Construction of SjAPI expression vectors

*Ascaris*-type peptide SjAPI is a novel animal toxin with the unique characteristic of five disulfide bridges. Therefore, we attempted to use two expression vectors, pET-28a and pGEX-4T-1, to produce recombinant SjAPI in *E. coli*. The cDNA sequence of SjAPI from the *Scorpiops jendeki* venom gland cDNA library was used as the template for the generation of fragments using polymerase chain reaction (PCR). The PCR product of SjAPI was digested with BamHI and XhoI, and inserted into a modified pET-28a expression vector. After confirmation by sequencing, the plasmid was transformed into *E. coli* Rosetta (DE3) cells for expression. The expression vector pGEX-4T-SjAPI was constructed and transformed by a method similar to that described previously [23].

### Site-directed mutagenesis

The QuikChange Site-Directed Mutagenesis Kit (Stratagene, U.S.A.) was used to produce mutants based on the wild-type plasmid pET-28a-SjAPI. All mutant plasmids were verified by DNA sequencing before expression.

### Expression and purification of SjAPI and its mutants

The expression of GST-SjAPI and purification of the recombinant SjAPI peptide were carried out according to the method previously described [23]. The expression and purification of the recombinant His-SjAPI peptide and its mutants was carried out as follows. Transformed cells containing the expression plasmid pET-28a-SjAPI were cultured at 37°C in LB medium with 30 μg/ml kanamycin. Protein synthesis was induced by the addition of 5–10 mM isopropyl β-D-thiogalactoside (IPTG) when the optical density at 600 nm reached 0.8–1.0. After 4 hours of continued growth at 37°C, cells from 1 L culture were harvested by centrifugation. The cell pellet was resuspended in phosphate-buffered saline (PBS) buffer and lysed by sonication on ice. The recombinant SjAPI was exclusively accumulated in inclusion bodies. The insoluble inclusion bodies were washed twice with washing buffer (1–2% Triton X-100 in PBS), and denatured in 2 ml denaturation solution (6 M guanidinium-HCl, 0.1 M Tris-HCl pH 8.0, 1 mM EDTA, 30 mM reduced glutathione). Then, rSjAPI was reactivated by 100-fold dilution in renaturation solutions with three different pHs (0.2 M ammonium acetate at pH 7.0, 8.5, or 9.5 containing 0.2 mM oxidized glutathione and 0.5 M arginine) at 16°C for 24 h. The soluble material was then desalted and enriched using centrifugal filter devices (Sartorius Stedim Biotech, Germany, cutoff value >5 kDa). The renatured peptide was finally purified by high-pressure liquid chromatography (HPLC) on a C18 column (10 mm ×250 mm, 5 μm; Elite-HPLC, China) with a constant flow rate of 5 ml/min. Peaks were detected at a wavelength of 230 nm. The fraction containing recombinant SjAPI was collected manually and lyophilized immediately. The molecular mass of purified rSjAPI was further analyzed by matrix-assisted laser desorption-ionization time-of-flight mass spectrometry (MALDI-TOF-MS; Voyager-DESTR; Applied Biosystems).

The SjAPI mutants were produced using the same method, with the refolding pH at about 9.5. The secondary structures of SjAPI and its mutants were analyzed by circular dichroism (CD) spectroscopy. All samples were dissolved in water at a concentration of about 0.2 mg/ml. Spectra were recorded at 25°C over a wavelength range of 250 to 190 nm with a scan rate of 50 nm/min on a Jasco-810 spectropolarimeter. Each CD spectrum was obtained as an average of three scans after subtracting the blank spectrum of water.

### Structure modeling and molecular dynamics (MD) simulation

MD simulation was used to predict the putative active site of SjAPI as follows. The atomic structure of SjAPI was modeled using an *Ascaris*-type peptide AMCI-1 (PDB code 1CCV) as the template as previously described [24]. The structure of elastase was extracted from the C/E-1 and elastase complex (PDB code: 1EAI) [16]. Then an SjAPI-elastase complex was obtained by the distance restraint homologous modeling method on the basis of the C/E-1-elastase complex and subjected to MD simulation in explicit solvent to test its stability [25]. The structure of the SjAPI-α-chymotrypsin complex was obtained using a similar method on the basis of the guamerin-α-chymotrypsin complex (PDB code: 3BG4) [26].

### Serine protease inhibitory activity assay

The inhibitory activities of SjAPI and its mutants were tested by methods similar to those described previously [9], [25]. Trypsin (bovine pancreatic trypsin; EC 3.4.21.4), chymotrypsin (bovine pancreatic α-chymotrypsin; EC 3.4.21.1), elastase (porcine pancreatic elastase; EC 3.4.21.36), and their respective chromogenic substrates Na-benzoyl-L-arginine 4-nitroanilide hydrochloride N-succinyl-Ala-Ala-Pro-Phe-p-nitroanilide, and N-succinyl-Ala-Ala-Ala-p-nitroanilide, were purchased from Sigma (U.S.A). The initial rate of every reaction was monitored continuously at 405 nm for 5 min at 25°C. The inhibitory constant (Ki) of the protease/inhibitor complex was determined by Lineweaver-Burk plots followed by further slope replotting to yield a Ki value.

## Results

### Scorpion venom gland cDNA libraries, a new source of *Ascaris*-type peptides

**Figure 1:**
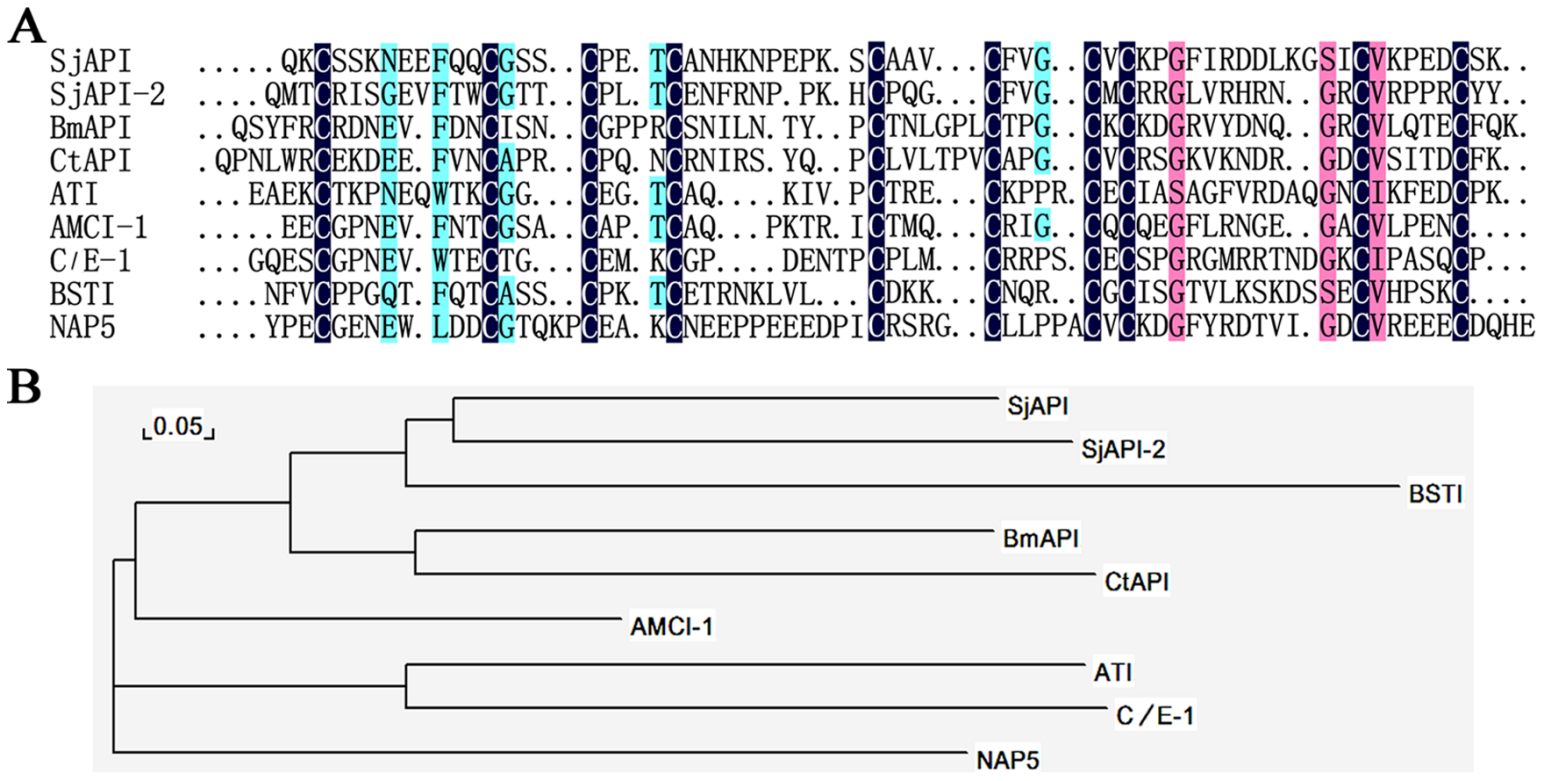
Members of the first *Ascaris*-type animal toxin family. (A) Amino acid sequence alignments of SjAPI, SjAPI-2, CtAPI, and BmAPI with known representative *Ascaris*-type peptides. The sequence identities of different *Ascaris*-type peptides to SjAPI were shown on the right side. (B) A minimum evolution (ME) tree of representative *Ascaris*-type proteins based on multiple sequence alignment.

From the sequenced ESTs of scorpion cDNA libraries, we identified four genes encoding the *Ascaris*-type peptides SjAPI, SjAPI-2, CtAPI, and BmAPI (Fig. 1A). Multiple sequence alignments showed that the four *Ascaris*-type peptides from scorpion venom share homology with typical *Ascaris*-type venom peptides from other animals, such as ATI from *Ascaris lumbricoides* var. *suum* [27], AMCI-1 from *Apis mellifera* [18], BSTI from the skin secretions of *Bombina bombina* [17], C/E-1 from *Ascaris suum* [16], and NAP5 from *Ancylostoma caninum* [28]. Phylogenetic tree analysis showed that among the representative *Ascaris*-type peptides, four *Ascaris*-type peptides from scorpion venom (SjAPI, SjAPI-2, CtAPI, BmAPI) and one *Ascaris*-type peptide from the secretion of frog skins (BSTI) were assigned to a single group (Fig. 1B), suggesting similarities between peptide toxins from animal venom glands and secreted peptides from amphibious skins [11].

### Sequence analysis of SjAPI, a member of the *Ascaris*-type toxin family

**Figure 2:**
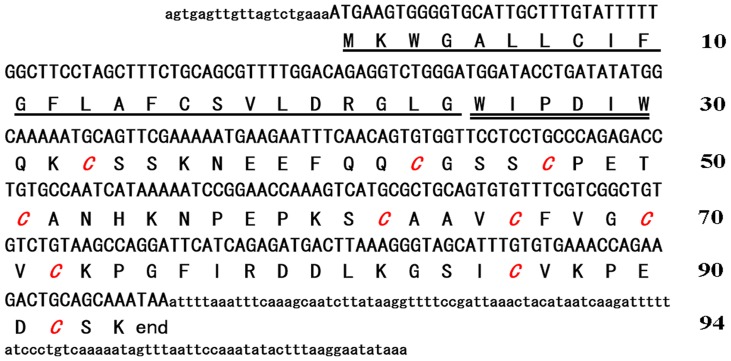
Precursor nucleotide sequence and deduced amino acid sequence of *Ascaris*-type toxin SjAPI. The signal peptide, propeptide, and mature peptide were marked and the cysteine residues of the mature peptide were shown in italics.

As a representative member of the *Ascaris*-type toxin family, SjAPI has a precursor nucleotide sequence of 415 nucleotides (nt) including three parts: the 5′ untranslated region (UTR), the ORF, and the 3′ UTR. The 5′ UTR is only 20 nt long and the 3′ UTR is 110 nt long. The ORF region of 285 nt encodes a precursor polypeptide of 94 amino acid residues, including a signal peptide of 24 residues, a short propeptide of 6 residues, and a mature peptide of 64 residues, with the unique feature of 10 cysteine residues (Fig. 2). The mature SjAPI peptide possesses the classical *Ascaris*-type cysteine framework reticulated by five disulfide bridges, which is conserved in classical *Ascaris*-type peptides such as ATI and AMCI-1 and is distinct from all known protease inhibitors from venomous animals [11], [18], [27].

### Cloning, expression, and refolding of SjAPI

**Figure 3:**
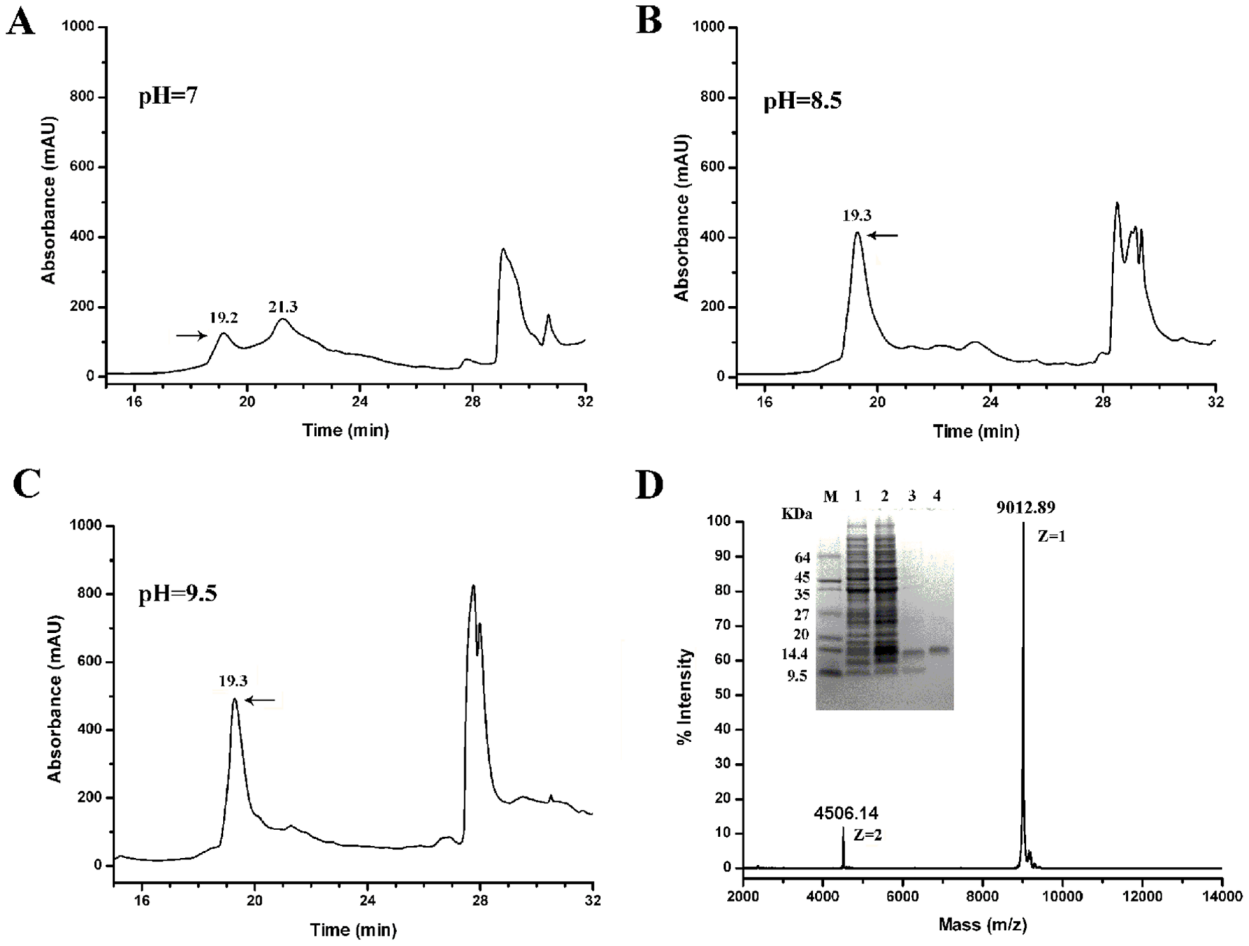
Purification and determination of recombinant *Ascaris*-type toxin SjAPI. (A) Purification of rSjAPI refolded at pH 7.0 by RP-HPLC. (B) Purification of rSjAPI refolded at pH 8.5 by RP-HPLC. (C) Purification of rSjAPI refolded at pH 9.5 by RP-HPLC. (D) Tricine-SDS-PAGE and MALDI-TOF-MS mass spectrum analysis of rSjAPI purification. M, marker; lane 1, total cell-free extract of *E. coli* carrying pGEX-6p-1-ImKTx1 uninduced; lane2, total cell-free extract of *E. coli* carrying pGEX-6p-1-ImKTx1 induced with IPTG; lane 3, purified rSjAPI peptide using centrifugal filter; 4, purified rSjAPI by RP-HPLC. MALDI-TOF-MS showed a singly charged ion at *m/z* 9012.89.

Two expression systems were attempted to obtain recombinant SjAPI. Small-scale expression tests showed that both GST-SjAPI and His-SjAPI were expressed in inclusion bodies, so we chose His-SjAPI to produce large quantities of recombinant SjAPI peptide. Refolding of *Ascaris*-type peptides was a challenge due to their 10 cysteine residues [29], and we chose to refold SjAPI at three different pH levels in the present of 0.5 M arginine (Fig. 3A–C). Soluble and functional SjAPI was successfully obtained upon refolding at pH 9.5. Recombinant SjAPI (rSjAPI) was separated by reverse phase (RP)-HPLC and the peak eluting at 19.3 min, corresponding to rSjAPI, was collected manually. MALDI-TOF-MS showed a doubly charged ion at *m/z* 4506.14 and a singly charged ion at *m/z* 9012.89. Both MALDI-TOF-MS and sodium dodecyl sulfate polyacrylamide gel electrophoresis (SDS-PAGE) analyses showed that rSjAPI was expressed and purified successfully (Fig. 3D). Subsequently, rSjAPI was quantified by the BCA Protein Assay kit (Thermo Fisher Scientific) and stored at −20°C after freeze-drying.

### Pharmacological activities of rSjAPI on serine proteases

**Figure 4:**
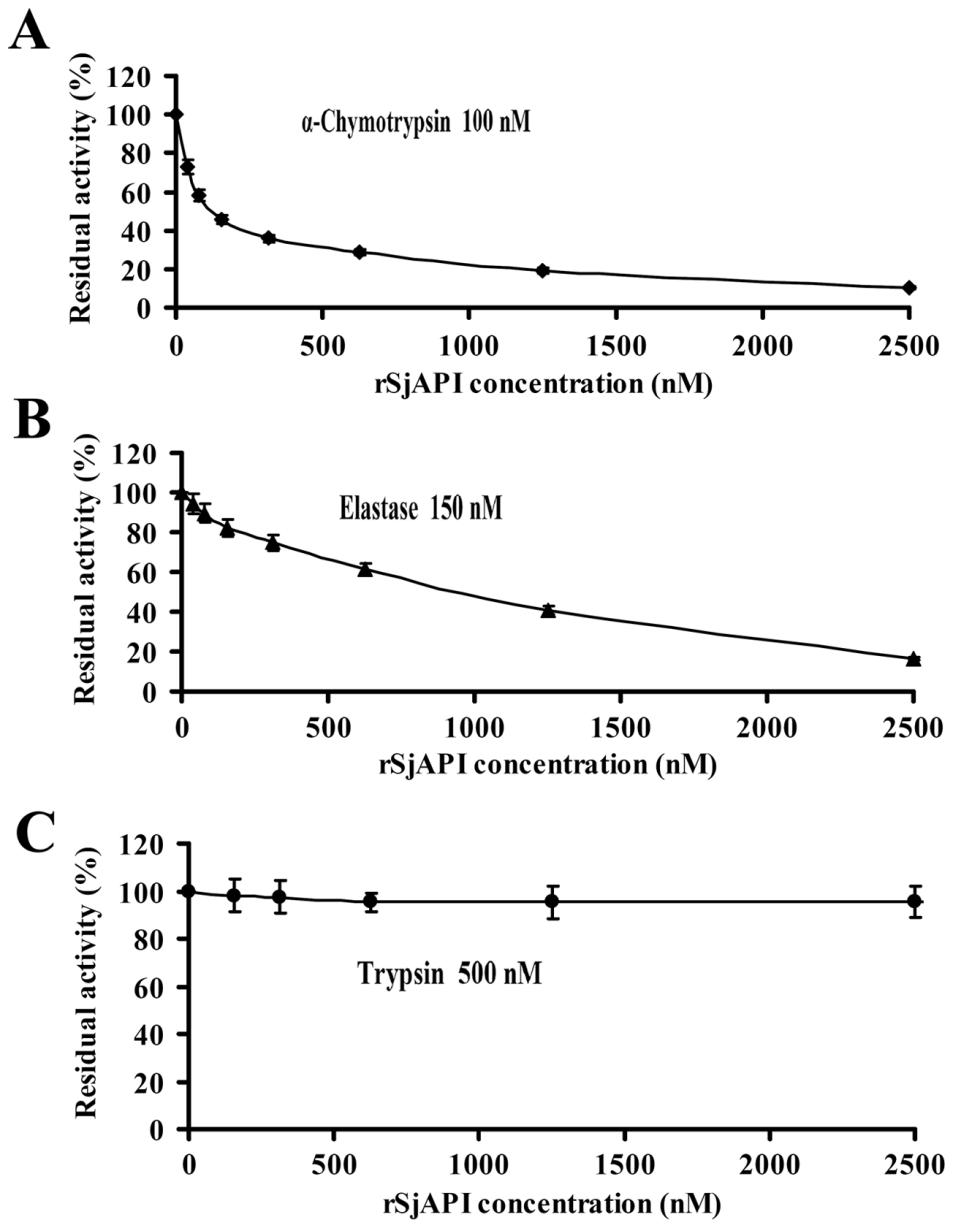
Serine protease inhibitory activities of rSjAPI with different concentrations. (A) The concentration dependence of inhibitions on trypsin was shown with different concentrations of rSjAPI. (B) The concentration dependence of inhibitions on α-chymotrypsin was shown with different concentrations of rSjAPI. (C) The concentration dependence of inhibition on elastase was shown with different concentrations of rSjAPI. Trypsin (final concentration 500 nM), α-chymotrypsin (final concentration 100 nM), elastase (final concentration 150 nM) were each incubated with various concentrations of rSjAPI (0–2500 nM) for 30 min. All data represent the mean ± standard error of at least three experiments.

**Figure 5:**
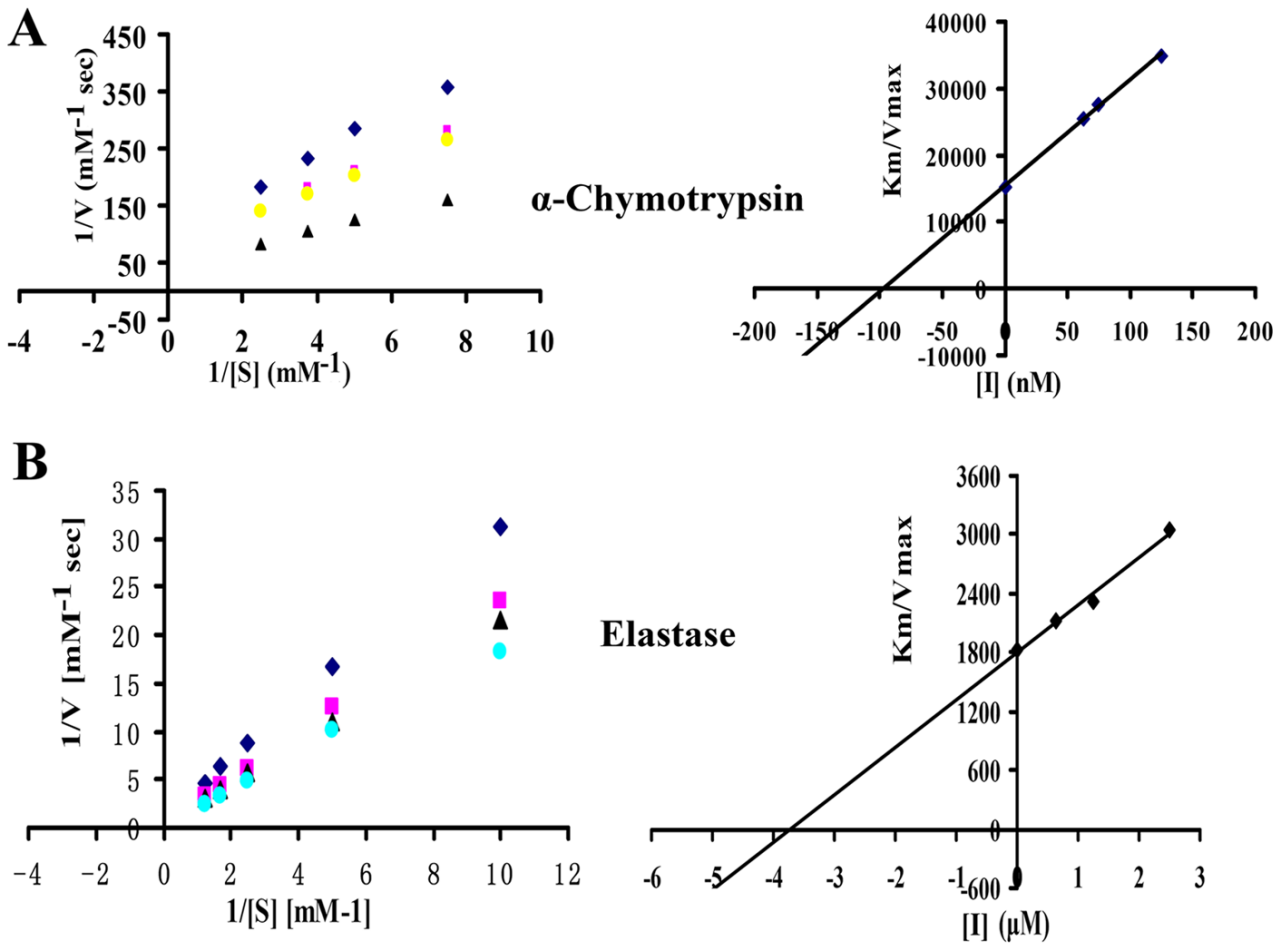
The Ki values of rSjAPI with α-chymotrypsin and elastase. (A) Lineweaver-Burk plots for the determination of Km/Vmax values of α-chymotrypsin activity on a synthetic chromogenic substrate in the absence or presence of 60 nM, 75 nM, and 125 nM rSjAPI, respectively. Secondary plot: the slopes (Km/Vmax) of the primary Lineweaver-Burk graphs were plotted against the inhibitor concentration. The inhibitory constant (Ki) is determined from the intercept point on the x-axis. (B) Lineweaver-Burk plots and secondary plots show the inhibition of elastase by rSjAPI in the absence or presence of 625 nM, 1250 nM, and 2500 nM rSjAPI, respectively. Ki values were showed as the mean ± standard error at least three experiments.

After obtaining the rSjAPI peptide, we tested its inhibitory effects on three representative serine proteases, trypsin at 500 nM, α-chymotrypsin at 100 nM and elastase at 150 nM. The results showed that rSjAPI was a dual-functional peptide with α-chymotrypsin- and elastase-inhibiting properties, but had no effect on trypsin (Fig. 4). The inhibitory constant (Ki) was further determined by Lineweaver-Burk plots and subsequent slope replotting. rSjAPI inhibited α-chymotrypsin and elastase with Ki values of 97.1±6.5 nM and 3.7±0.4 μM, respectively (Fig. 5 and Table 1).

**Table 1:** Serine protease inhibitors from animal venoms.

Source	Name	Trypsin (M)	Chymotrypsin (M)	Elastase (M)	Ref
*Kunitz-type*					
Snake	Bungaruskunin	9.8×10^−4^	6.1×10^−6^	6.9×10^−4^	10
Snake	OH-TCI	3.9×10^−7^	8.5×10^−8^	/	38
Sea anemone	APEKTx1	1.2×10^−7^	/	/	39
Spider	HWTX-XI	2.3×10^−10^	/	/	40
Scorpion	SdPI	1.6×10^−7^	/	/	25
*Ascaris-type*					
Scorpion	SjAPI	/	9.7×10^−8^	3.7×10^−6^	This work

Ki values of representative animal toxins with serine protease inhibiting activities were listed.

### Functional site analysis and chimeras design of SjAPI

**Figure 6:**
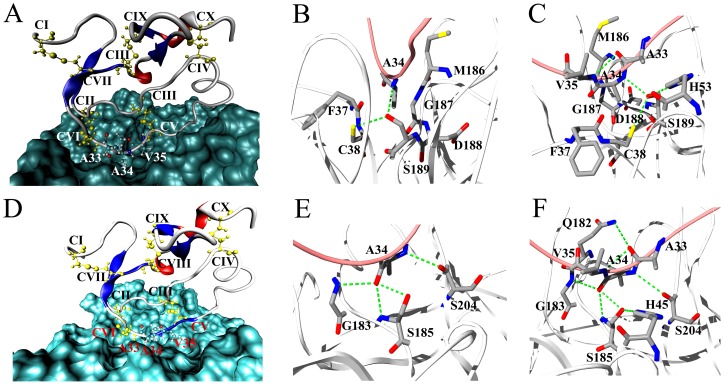
Molecular simulation of SjAPI interacting with chymotrypsin and elastase. (A) The SjAPI-chymotrypsin complex predicted by MD simulation. (B) Ala34, the P1 site of SjAPI, fits into the pocket of chymotrypsin. (C) Residues of chymotrypsin interacting with Ala33, Ala34 and Val35 residues, the P1′, P1 and P2 sites of SjAPI. (D) The SjAPI-elastase complex predicted by MD simulation. (E) Ala34, the P1 site of SjAPI, fits into the pocket of elastase. (F) Residues of elastase interacting with Ala33, Ala34 and Val35 residues, the P1′, P1 and P2 sites of SjAPI.

Structure-function relationship research has shown that the binding loop of *Ascaris*-type peptides is located in a conserved region between cysteine V and VI [30]. In SjAPI, this region corresponds to the sequence “AAV,” in which Ala34 is the P1 site, Ala33 is the P2 site, and Val35 is P1' site (Fig. 1A and Fig. S1). MD simulation was employed to probe inhibitor-protease interactions in detail. The results confirmed that the inhibitory activity of SjAPI was more potent for chymotrypsin than for elastase (Fig. 6). Beside the hydrophobic interactions, the amide group of Ala34 in SjAPI and the hydroxyl group of Ser189 in chymotrypsin formed hydrogen bond pair and contributed to the interaction between SjAPI and chymotrypsin (Fig. 6B and C). The carboxyl group of Ala34 in SjAPI formed hydrogen bond pairs with the groups of Ser185 and Gly183 in elastase, and the carboxyl group of Ala33 in SjAPI formed hydrogen bond pair with the group of Gln185 in elastase, and all these bond pairs contributed to the interaction between SjAPI and elastase (Fig. 6E and F).

**Figure 7:**
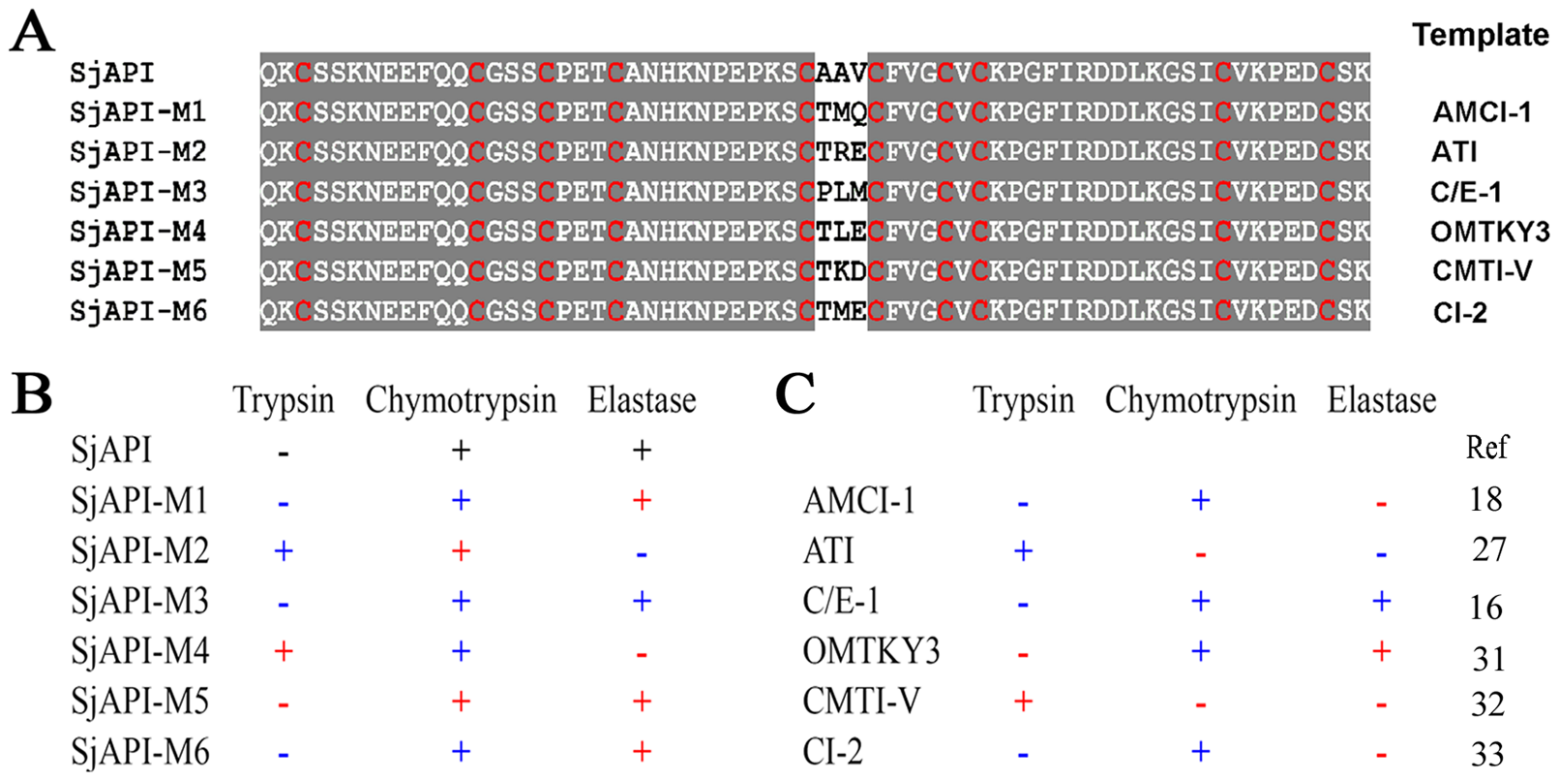
The six designed chimeras that transfer the active sites of different peptides from *Ascaris*, Kazal, and potato I family folds. (A) Amino acid sequence alignments of six chimeras and SjAPI. SjAPI-M1 was from the *Ascaris*-type peptide AMCI-1, SjAPI-M2 was from the *Ascaris*-type peptide ATI, SjAPI-M3 was from the *Ascaris*-type peptide C/E-1, SjAPI-M4 was from the Kazal-type peptide OMTKY3, SjAPI-M5 was from the potato I family peptide CMTI-V, and SjAPI-M6 was from the potato I family peptide CI-2. (B) Comparison of the serine protease inhibitory activity profiles of six chimeras with those of their templates and SjAPI.

Considering that the “AAV” sequence is made up of three short side chain residues, we designed six chimeras to further evaluate the functional sites P2, P1, and P1' of SjAPI instead of using conventional alanine-scanning-mutagenesis [25]. The chimeras included SjAPI-M1 from the *Ascaris*-type peptide AMCI-1 [18], SjAPI-M2 from the *Ascaris*-type peptide ATI [27], SjAPI-M3 from the *Ascaris*-type peptide C/E-1 [16], SjAPI-M4 from the Kazal-type peptide OMTKY3 [31], SjAPI-M5 from the potato I family peptide CMTI-V [32], and SjAPI-M6 from the potato I family peptide CI-2 [33] (Fig. 7A). CD spectroscopy analysis showed that the six chimeras had secondary structures similar to that of SjAPI (Fig. S2). Enzyme and inhibitor reaction kinetics experiments showed that all six chimeras were effective serine protease inhibitors, but exhibited different activities corresponding to their different P2, P1, and P1' site residues (Fig. S3).

As a unique dual serine protease inhibitor with three short side chain residues (“AAV”) at the P1, P2, and P1' sites, SjAPI was a good molecular scaffold to study the relationship between P1, P2, P1' sites and other potent sites in serine protease inhibitors. Our results showed that the inhibitory activity profiles of the six chimeras were not always consistent with their templates, although the P1, P2, and P1' sites were the same as in their templates (Fig. 7B). For example, the chimera SjAPI-M4 and the wild-type peptide OMTJY3 have the same P2, P1, and P1' sites, “TLE.” However, enzyme and inhibitor reaction kinetics experiments showed that SjAPI-M4 inhibits trypsin and α-chymotrypsin, but has no effect on elastase, in contrast to the wild-type peptide OMTJY3, which shows α-chymotrypsin and elastase inhibitory properties. The chimera SjAPI-M2 and the wild-type peptide ATI have the same P2, P1, and P1' sites “TMQ,” but SjAPI-M2 can inhibit α-chymotrypsin, whereas ATI has no effect on α-chymotrypsin. On the other hand, SjAPI-M1, SjAPI-M3, SjAPI-M5 and SjAPI-M6 retained inhibitory activity profiles that were partly similar to that of SjAPI, although their P2, P1, and P1' sites were completely different. SjAPI-M2 and SjAPI-M4 retained the α-chymotrypsin inhibitory activity and lost the elastase inhibitory activity of SjAPI, but the acquired new trypsin inhibitory activity. These results indicate that residues other than those at the P2, P1, and P1' sites might also play important roles in inhibitor-protease interactions and should be considered in protease inhibitor engineering to improve their activities and specificities [34], [35].

## Discussion

Over 400 million years, scorpion evolved a combinatorial library of bioactive components including peptides, proteins, and other biologically active substances [36] In this work, by screening scorpion venom gland cDNA libraries, we identified the first *Ascaris*-type animal toxin family, and characterized a dual-function *Ascaris*-type peptide SjAPI with α-chymotrypsin- and elastase-inhibitory activities.

Overall, our present work has provided the first insight into the following unique structural and functional features of the *Ascaris*-type animal toxin family: (1) *Molecular diversity of Ascaris-type toxins*: In this study, we identified four *Ascaris*-type peptides from scorpion venom glands, and all four *Ascaris*-type peptides share low homology with known *Ascaris*-type peptides from other phyla. Given a large number of unidentified toxin peptides from scorpion (over 90%) and other venomous animals, such as snake, spider, snail, and sea anemone [37], our results suggested that animal venom glands might be a new source of *Ascaris*-type peptides, and enriched the diversity of *Ascaris*-type peptides. (2) *Structural diversity of venomous animal serine protease inhibitors*: The three-dimensional (3-D) structure of SjAPI was built using AMCI-1 (PDB code 1CCV) as a template and showed a typical *Ascaris*-type scaffold (Fig. S1) [18]. Prior to our work, all known serine protease inhibitors from venomous animals were *Kunitz*-type peptides, such as Hg1 from scorpion [19], OH-TCL and Bungaruskunin from snake [10], [38], APEKTx1 from sea anemone [39], and HWTX-XI from spider [40]. Our study is the first to identify *Ascaris*-type peptide toxins with serine protease-inhibiting activities, thereby indicating the structural diversity of venomous animal serine protease inhibitors and also enriching diverse structural folds of scorpion toxins (Fig. S4). (3) *Functional diversity of venomous animal serine protease inhibitors*: Animal toxins must withstand a series of serine protease tests, and the diverse functions of different serine protease inhibitors might be needed in animal venoms [10], [11], [40]. In our previous work, we identified scorpion derived protease inhibitor (SdPI), and it inhibits trypsin, but not α-chymotrypsin and elastase. Our prevent work showed that SjAPI inhibited α-chymotrypsin and elastase, but not trypsin, which indicated that the functional diversity and complementarity of serine protease inhibitors from animal venoms (Table 1). (4) *Unique molecular template to produce diverse serine protease inhibitors*: SjAPI contains the unique functional residues “AAV” at the P2, P1, and P1' sites. Our primary chimera designs indicated that all six chimeras were effective inhibitors of different serine proteases, but had different activities and specificities. Considering the difficulties in producing cysteine-enriched peptides and the functional expression of wild-type SjAPI and mutants in *E. coli* at about 2 mg/ml, our work provides an effective strategy for the design and production of various serine protease inhibitors using SjAPI as template [35], [41].

## Conclusion

In summary, we identified the first *Ascaris*-type animal toxin family and characterized the unique *Ascaris*-type toxin SjAPI that has dual serine protease-inhibiting properties. To our knowledge, SjAPI is the first functionally characterized *Ascaris*-type toxin derived from animal venoms and represents a new structural fold that has been recruited into animal venoms [11].

## Supporting Information

Figure S1 **Modeling of the 3-D structure of SjAPI.** (A) The 3-D structure of SjAPI and the respective active residues were labeled. (B) The structure assessment for the modeled SjAPI displayed in the Ramachandran plot.
(TIF)

Figure S2 **Circular dichroism spectrum analyses of rSjAPI and its chimeras.** The six chimeras are SjAPI-M1, SjAPI-M2, SjAPI-M3, SjAPI-M4, SjAPI-M5, and SjAPI-M6. The measurement was carried out in the UV wavelength range of 250–190 nm at 25°C in water on a Jasco-810 spectropolarimeter at a concentration of about 0.2 mg/ml. All data represent the mean of at least three experiments.
(TIF)

Figure S3 **Serine protease inhibitory activities of six chimeras.** (A) The concentration dependence of inhibitions on trypsin was shown using different concentrations of SjAPI and six chimeras. (B) The concentration dependence of inhibitions on elastase was shown using different concentrations of SjAPI and six chimeras. (C) The concentration dependence of inhibitions on α-chymotrypsin was shown with different concentrations of SjAPI and six chimeras (0–2500 nM).
(TIF)

Figure S4 **Diverse structural folds of scorpion toxins.** (A) Representative scorpion toxin charybdotoxin with CSα/β fold. (B) Representative scorpion toxin κ-Hefutoxin 1 with CSα/α fold. (C) Representative scorpion toxin Maurocalcine with ICK fold. (D) Representative scorpion toxin U1-LITX-Lw1a with DDH fold. (E) Representative scorpion toxin SdPI with *Kunitz-type* fold. (F) Representative scorpion toxin SjAPI with *Ascaris-type* fold.
(TIF)
