# Supplementary figures and images for: SjAPI, the First Functionally Characterized Ascaris-Type Protease Inhibitor from Animal Venoms
Source: PLoS One. 2013 Mar 22;8(3):e57529. doi: 10.1371/journal.pone.0057529 (PMC3606364; doi:10.1371/journal.pone.0057529)

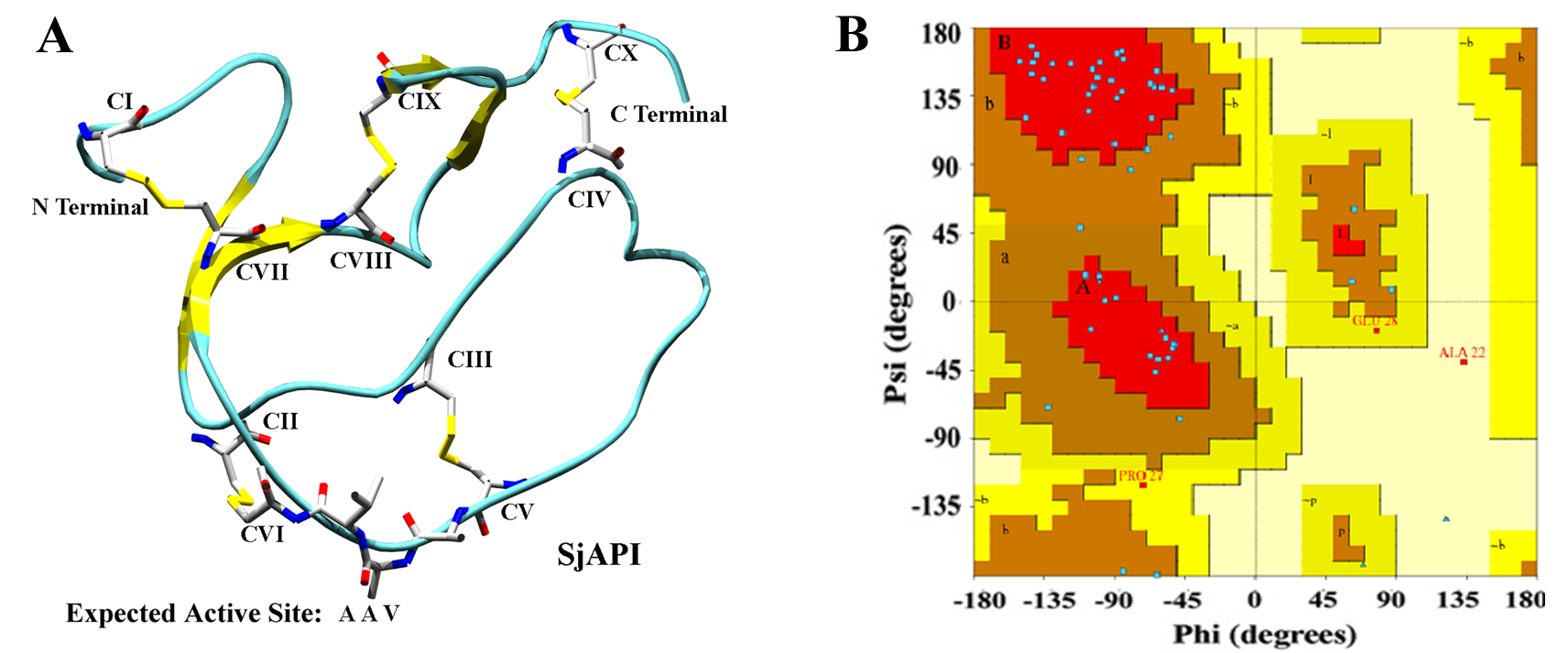

Supplement: Figure S1 — Modeling of the 3-D structure of SjAPI. (A) The 3-D structure of SjAPI and the respective active residues were labeled. (B) The structure assessment for the modeled SjAPI displayed in the Ramachandran plot. (TIF) [file pone.0057529.s001.tif]

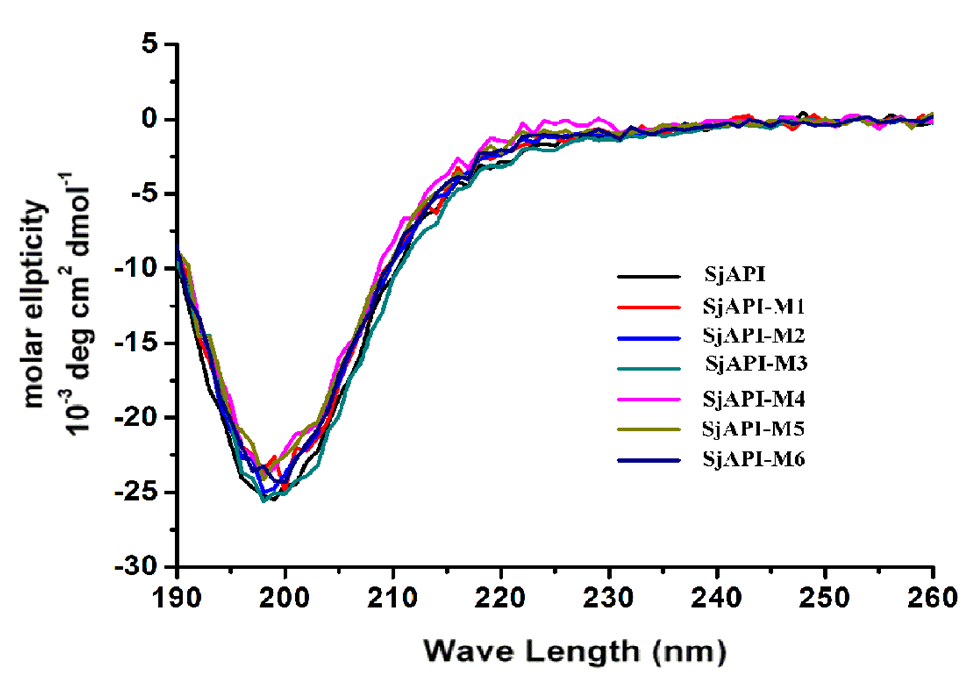

Supplement: Figure S2 — Circular dichroism spectrum analyses of rSjAPI and its chimeras. The six chimeras are SjAPI-M1, SjAPI-M2, SjAPI-M3, SjAPI-M4, SjAPI-M5, and SjAPI-M6. The measurement was carried out in the UV wavelength range of 250–190 nm at 25°C in water on a Jasco-810 spectropolarimeter at a concentration of about 0.2 mg/ml. All data represent the mean of at least three experiments. (TIF) [file pone.0057529.s002.tif]

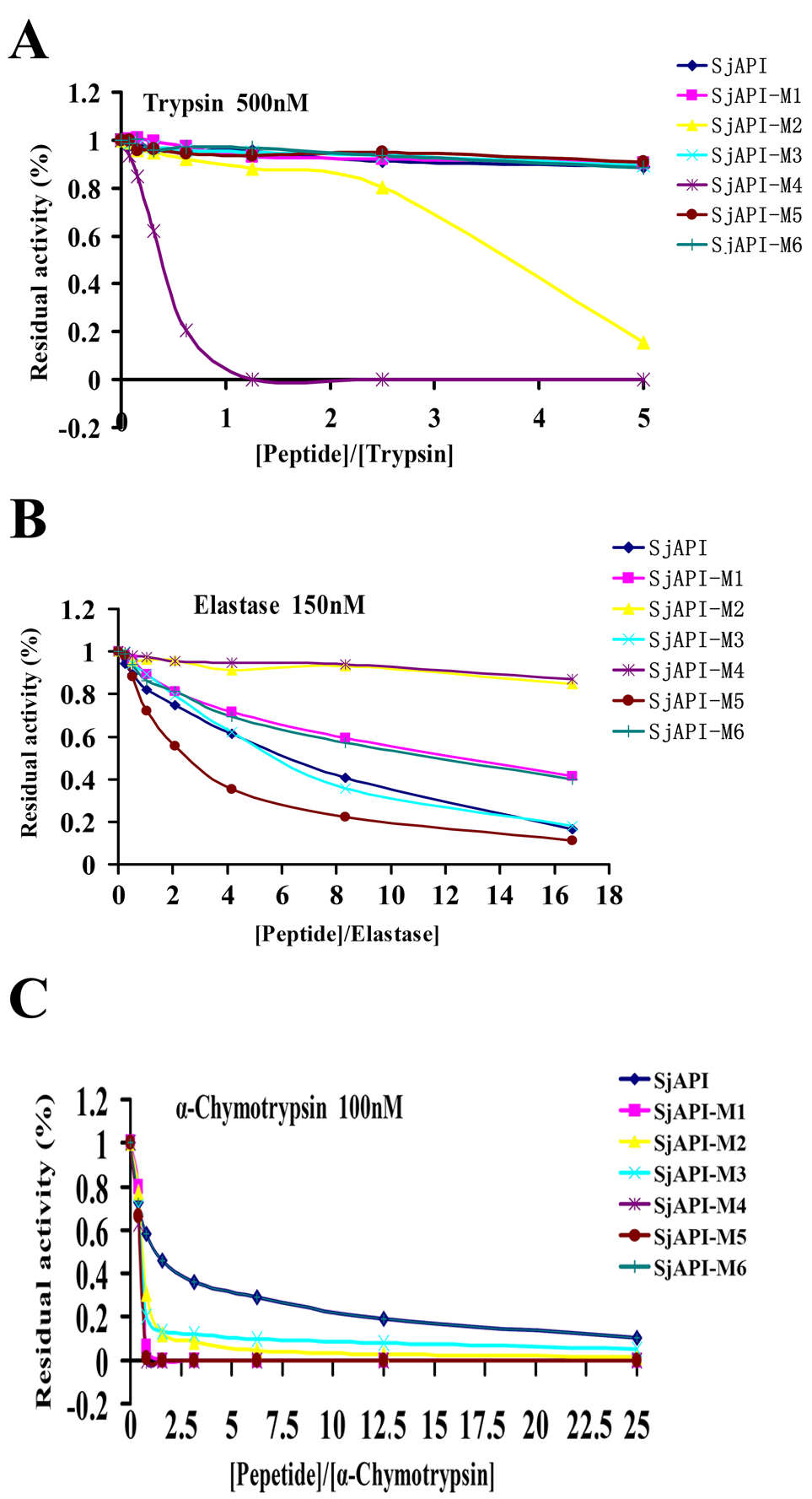

Supplement: Figure S3 — Serine protease inhibitory activities of six chimeras. (A) The concentration dependence of inhibitions on trypsin was shown using different concentrations of SjAPI and six chimeras. (B) The concentration dependence of inhibitions on elastase was shown using different concentrations of SjAPI and six chimeras. (C) The concentration dependence of inhibitions on α-chymotrypsin was shown with different concentrations of SjAPI and six chimeras (0–2500 nM). (TIF) [file pone.0057529.s003.tif]

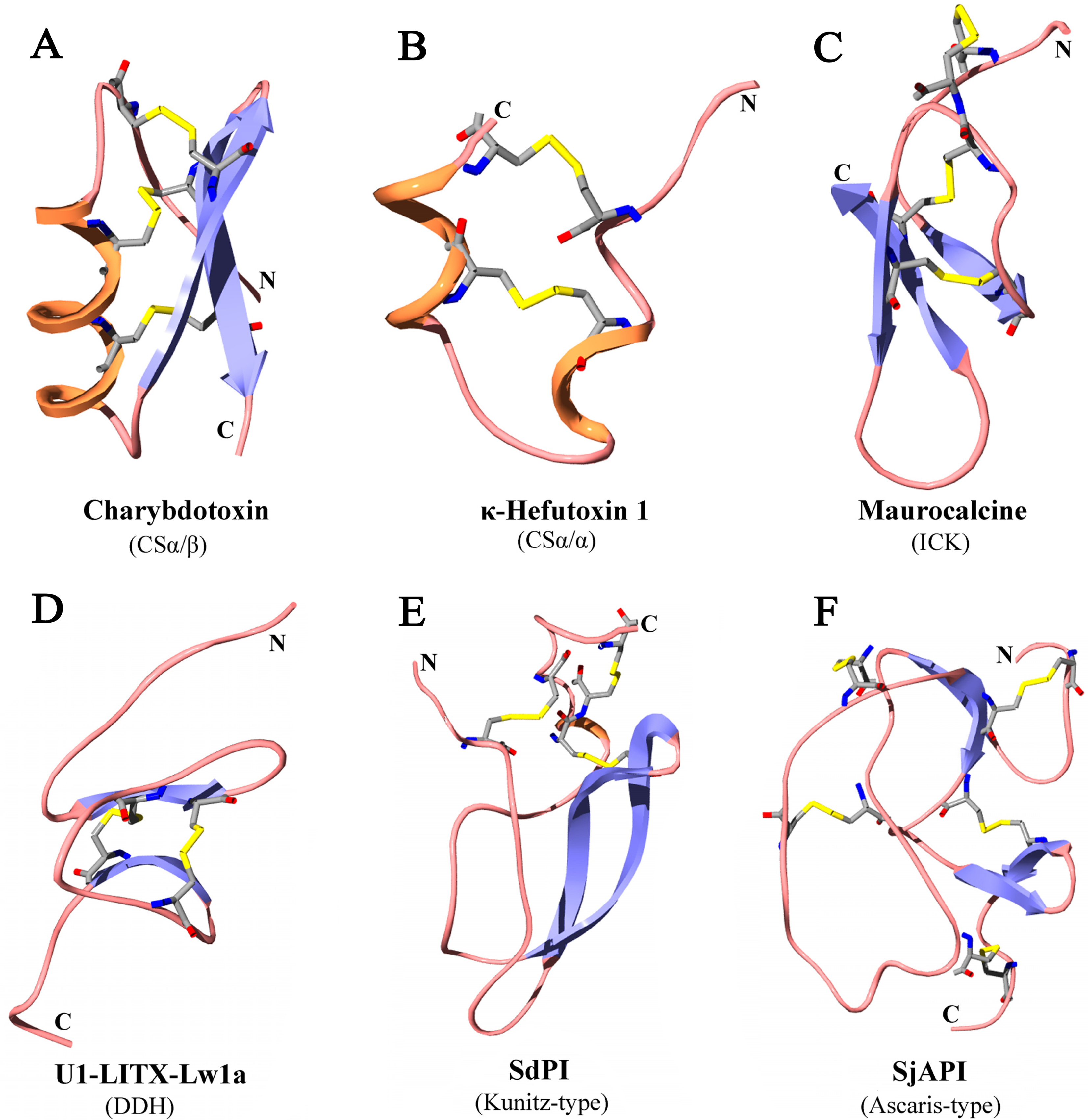

Supplement: Figure S4 — Diverse structural folds of scorpion toxins. (A) Representative scorpion toxin charybdotoxin with CSα/β fold. (B) Representative scorpion toxin κ-Hefutoxin 1 with CSα/α fold. (C) Representative scorpion toxin Maurocalcine with ICK fold. (D) Representative scorpion toxin U1-LITX-Lw1a with DDH fold. (E) Representative scorpion toxin SdPI with Kunitz-type fold. (F) Representative scorpion toxin SjAPI with Ascaris-type fold. (TIF) [file pone.0057529.s004.tif]
